# Impact of an Anti-infection Double Lumen Bundle on the incidence of central venous catheter–related infections in haematological patients: a retrospective cohort study

**DOI:** 10.1016/j.infpip.2026.100536

**Published:** 2026-03-30

**Authors:** M. Lazarevic Lindblad, E. Pålsson, V. Lazarevic, O. Borgquist, T. Kander

**Affiliations:** a Department of Clinical Sciences, Lund University, Lund, Sweden; b Department of Intensive and Perioperative Care, Skåne University Hospital, Lund, Sweden; c Department of Haematology, Oncology and Radiation Physics, Skåne University Hospital, Lund, Sweden; d Department of Cardiothoracic Surgery, Anaesthesia and Intensive Care, Skåne University Hospital, Lund, Sweden

**Keywords:** Central venous catheter, Catheter-related infection, Catheter bundle, Haematology, Infection prevention

## Abstract

**Background:**

Central venous catheters (CVCs) are commonly used in patients with haematological diseases but are associated with infectious complications. The CVC Anti-infection Double Lumen Bundle was introduced in December 2020 to reduce this risk. The bundle included a double-lumen noble metal alloy–coated CVC and chlorhexidine-impregnated dressings. This study evaluated whether the bundle was associated with a reduction in catheter-related infections.

**Methods:**

Non-tunnelled CVC insertions with a dwell time ≥24 h in adults treated for haematological diseases between May 2013 and June 2024 were included. Data were extracted from the electronic health records. The main objectives of the study were to investigate the proportions of suspected catheter-related infection (sCRI) and catheter-related bloodstream infection (CRBSI). Secondary objectives were catheter tip colonization and incidence of sCRI and CRBSI per 1000 catheter-days.

**Results:**

A total of 907 CVC insertions in 690 patients were analysed (471 before and 436 after the bundle implementation date). No differences were observed in the proportions of sCRI (6.4% vs 7.6%), CRBSI (0.4% vs 0.7%) or catheter tip colonization (4.7% vs 4.4%). The incidence per 1000 catheter-days also did not differ (sCRI: 1.67 vs 2.34 and CRBSI: 0.11 vs 0.21). In multi-variable analysis, no variables were associated with a higher risk of sCRI, whereas antibiotic administration at insertion was associated with a lower risk.

**Conclusion:**

The introduction of the Anti-infection Double Lumen Bundle did not reduce CVC-related infectious complications in patients with haematological diseases.

## Introduction

Patients with haematological diseases often require a central venous catheter (CVC) to facilitate their treatment. Unfortunately, these patients are highly susceptible to CVC-related infectious complications, with a reported incidence ranging between 0.64 and 7.6 per 1000 catheter-days [[1], [2], [3]]. Catheter-related infections result in prolonged hospital stay, increased costs and mortality, as well as extended patient suffering [4,5]. To reduce infectious complications, CVCs coated with a noble metal alloy have been developed. This has been shown to inhibit bacterial adhesion and biofilm formation compared with uncoated catheters [6]. In addition, a randomized controlled trial demonstrated a 52% reduction in catheter-related infections using this technology [7].

Chlorhexidine-impregnated dressings covering the catheter insertion site have been investigated as a strategy to reduce catheter-related infections. However, most evidence derives from heterogeneous patient populations, and studies in neutropenic patients have shown inconsistent results [[8], [9], [10], [11]].

Current haematology–oncology guidelines emphasize standard infection prevention measures such as strict aseptic insertion technique, while evidence supporting additional measures such as antimicrobial-coated CVCs or chlorhexidine-impregnated dressings in this patient population remains limited [12].

A “CVC Anti-infection Double Lumen Bundle” was introduced in the Department of Haematology at Skåne University Hospital in Lund, Sweden, in December 2020. The bundle consisted of replacing the previously used single-lumen polyurethane CVC with a noble metal alloy–coated double-lumen CVC and introducing a chlorhexidine gluconate dressing instead of a standard transparent film dressing.

This retrospective before-and-after study examined whether the CVC Anti-infection Double Lumen Bundle reduced suspected catheter-related infections (sCRIs) and catheter-related bloodstream infections (CRBSIs). Secondary objectives were catheter tip colonization and risk factors for CVC-associated infection.

## Methods

The study was approved by the Swedish Ethical Review Authority (Dnr 2018/295), which waived the requirement for informed consent. The manuscript was prepared in accordance with STROBE (Strengthening the Reporting of Observational Studies in Epidemiology) guidelines for observational studies. Patients were not involved in the design, conduct, reporting or dissemination of the study. The findings are intended to support clinical decision-making and ongoing quality assurance.

All non-tunnelled CVC insertions in patients aged ≥18 years admitted to a haematological ward at Skåne University Hospital, Lund, Sweden, between 28^th^ May 2013 and 30^th^ June 2024 were included if catheter dwell time was ≥24 h. Insertions with missing removal dates were excluded. Infectious complications were compared before and after introduction of the CVC Anti-infection Double Lumen Bundle on 1^st^ December 2020.

### The CVC Anti-Infection Double Lumen Bundle

Following bundle implementation, the previously used single-lumen polyurethane CVC was replaced with a noble metal (gold, silver and palladium) alloy–coated double-lumen CVC (Bactiguard® Infection Protection [BIP] CVC; Bactiguard AB, Tullinge, Sweden) that generates a galvanic effect in contact with blood, preventing microbe adhesion and biofilm formation. In addition, the standard transparent film dressing covering the catheter insertion site was replaced with a chlorhexidine gluconate–impregnated dressing (3M^TM^ Tegaderm^TM^ CHG Chlorhexidine Gluconate, Saint Paul, Minnesota, USA).

### CVC insertion, management and removal

CVC insertions were performed in accordance with regional guidelines, as previously described [13,14]. Needle passes were self-reported by the operator and defined as the number of passes through the skin. Standard oral prophylaxis given to patients with haematological diseases at risk did not change before or after the implementation date. Valacyclovir and acyclovir were used interchangeably, while posaconazole was primarily used in patients with acute leukaemia, whereas fluconazole was used in patients undergoing stem cell transplantation. In addition, patients with haematological diseases received a single prophylactic dose of rifampicin prior to the CVC insertion in accordance with local institutional guidelines in place at the time, until this practice was discontinued in 2019.

Catheter tip cultures were obtained if catheter infection was suspected. Before CVC removal, the insertion site was disinfected with chlorhexidine 5 mg/mL and allowed to air-dry. The distal 3–5 cm of the catheter was cut and submerged in a sterile culture tube. The catheter tip was transferred to broth and sonicated at the laboratory before semiquantitatively cultured on substrates chosen to optimize growth. Growth of >100 colony-forming units (cfu) was considered significant colonization of the catheter. Secretions around the insertion site were also sampled and sent for cultures.

In cases of low suspicion of infection, the catheter was occasionally left in place, with results from paired peripheral and catheter blood cultures obtained within 15 min of each other. Blood cultures were incubated until microbial growth for a maximum of five days and analysed using the BD BACTEC^TM^ FX system (Becton, Dickinson and Company, Franklin Lakes, New Jersey, USA). All detected micro-organisms were considered in the analysis.

### Data collection

A fully automated structured query language (SQL) script was used to extract data from the CVC insertion and care forms from the electronic health record (EHR) system (Melior™, Oracle Health, North Kansas City, Missouri, USA). Detailed data were then manually extracted from the EHR and merged to a Microsoft Excel 2016 (Version 16, Microsoft, Redmond, Washington, USA) database. Any uncertainties were jointly assessed within the research group.

### Outcomes and definitions

The primary outcome measures were the proportions of sCRI and CRBSI before and after the implementation date. Secondary outcomes were catheter tip colonization and the incidence of sCRI and CRBSI per 1000 catheter-days.

Catheter-related infections were defined according to previously published criteria used in studies of CVC-related complications in patients with haematological diseases and established CRBSI definitions [1,12,15]. Systemic inflammatory response syndrome (SIRS) was defined as the simultaneous presence of at least two out of the following three criteria: fever (a single temperature of ≥38.3 °C or, ≥38.0 °C sustained for at least 1 h, or <36 °C), heart rate >90 beats per minute and respiratory rate >20 breaths per minute. The white blood cell count usually included in SIRS was omitted due to the influence of ongoing haematological disease [16,17].

### Definition of suspected catheter-related infection (sCRI)

1. Clinical suspicion of a catheter-related infection, **and**
  ○ Either a positive catheter tip culture at removal or a positive peripheral blood culture obtained within 48 h before CVC removal, **and**
  ○ SIRS within 24 h prior to CVC removal,

**or**

2. Local catheter infection, defined as signs of local infection at the catheter insertion site within 72 h prior to CVC removal.

### Definition of CRBSI

CRBSI was defined as the presence of all of the following criteria:

1. Microbiological findings suggestive of catheter-related infection, defined as either
  ○ A positive catheter tip culture and a peripheral blood culture with the same micro-organism and antibiotic resistance pattern, obtained within 48 h prior to CVC removal, **or**
  ○ A ≥2-h earlier time to positivity in catheter blood compared with peripheral blood in paired blood cultures.
2. SIRS within 24 h prior to CVC removal.
3. No other likely or confirmed source of infection.

### Definition of catheter tip colonization

Catheter tip colonization was defined as microbial growth on the catheter surface with a positive catheter tip culture.

### Statistics and analyses

Sample size was based on the number of CVC insertions during the study period. CVC characteristics were reported for all insertions, whereas patient characteristics were reported once per patient (at the first CVC insertion). Data were presented as median and interquartile range (IQR) for continuous variables. Categorical and binary variables were presented as numbers and percentages. Baseline variables were considered potential independent variables for a multivariable regression model. Catheter tip cultures were obtained only when infection was clinically suspected. Catheters without a performed tip culture were therefore classified as negative in the analysis, reflecting the absence of clinical suspicion of catheter-related infection. sCRI, CRBSI and tip colonization before and after the bundle implementation date were tested using univariable regression analyses and were presented as odds ratios (ORs) with 95% confidence intervals (CIs). Outcomes were reported both as proportions per catheter insertion and as incidence per 1000 catheter-days to account for differences in catheter dwell time. The number of independent variables included in the multi-variable regression model was limited to approximately one variable per nine events to reduce the risk of model overfitting. Variables were selected based on previous studies and the results of the univariable analyses [18]. Model fit was evaluated using the Hosmer–Lemeshow test, and collinearity was assessed using Spearman correlation. Catheter dwell time, acute myeloid malignancy and insertion site were dichotomized in the multi-variable regression model in accordance with clinically established thresholds reflecting meaningful risk categories as well as observed baseline differences. A Poisson test was used to compare the incidence of sCRI and CRBSI per 1000 catheter-days. *P* values < 0.05 were considered statistically significant, and all tests were two-tailed.

Analyses were performed using R (version 4.5.2) together with RStudio (Version 2025.09.2+418, Posit PBC, Boston, Massachusetts, USA) using data from the original dataset.

## Results

A total of 907 CVC insertions in 690 patients were included. Of these insertions, 471 (52%) occurred before the implementation date and 436 (48%) after. Some patients received more than one CVC during the study period. Due to missing information regarding removal date, 67 CVCs (37 before vs 30 after the implementation date) were excluded. (None of them had signs of infectious complications.)

Baseline characteristics per catheter and per patient are presented in Tables I and II. Median dwell time was 28 days (IQR: 19–51) before and 25 days after bundle implementation (IQR: 17–38) (*P* = 0.002). The subclavian vein was the most common insertion site in both periods (86% vs 71%; *P* < 0.001), while the preferred insertion side shifted from left to right (39% vs 80%; *P* < 0.001). Most CVCs were single lumen before implementation (89%) but rarely thereafter (6%) (*P* < 0.001). The use of additional systemic antibiotics decreased from 73% to 40% (*P* < 0.001). BIP CVCs were used in 82% of insertions after implementation.

**Table I:** Baseline characteristics per catheter^a^

Baseline characteristics per catheter	Total	Before bundle implementation	After bundle implementation	*P*-value
	*N* = 907	*N* = 471	*N* = 436	
**Total CVC days**	32,126	18,001	14,125	-
**Catheter dwell time, days**	27 [18–43]	28 [19–51]	25 [17–38]	0.002
**Vein for insertion**				
Subclavian vein	709 (79)	401 (86)	308 (72)	<0.001
Internal jugular vein	182 (20)	66 (14)	116 (27)	<0.001
External jugular vein	3 (0.3)	0 (0)	3 (0.7)	NS
Missing	13	4	9	NS
**Side of insertion**				
Right	525 (59)	184 (39)	341 (80)	<0.001
Left	368 (41)	282 (61)	86 (20)	<0.001
Missing	14	5	9	NS
**Catheter model**				
BIP CVC^b^	345 (39)	0 (0)	345 (82)	<0.001
Non-coated^c^	543 (61)	466 (100)	77 (18)	<0.001
Missing	19	5	14	0.043
**Number of lumens**				
1	427 (49)	402 (89)	25 (5.8)	<0.001
2	426 (49)	30 (6.7)	396 (93)	<0.001
3	13 (1.5)	10 (2.2)	3 (0.7)	NS
5	12 (1.4)	8 (1.8)	4 (0.9)	NS
Missing	29	21	8	0.040
**Needle passes**				
1	630 (74)	306 (71)	324 (78)	0.012
2	132 (16)	76 (18)	56 (14)	NS
3	51 (6.0)	27 (6.2)	24 (5.8)	NS
≥4	33 (3.9)	24 (5.5)	9 (2.2)	0.019
Missing	61	38	23	NS
**Insertion method**				
Real-time ultrasound	743 (83)	320 (69)	423 (99)	<0.001
Other technique^d^	150 (17)	145 (31)	5 (1.2)	<0.001
Missing	14	6	8	NS
**Cause for removal**				
Ceased need	637 (70)	331 (70)	306 (70)	NS
Suspected infection	160 (18)	75 (16)	85 (19)	NS
Deceased patient	62 (6.8)	35 (7.4)	27 (6.2)	NS
Other^e^	48 (5.3)	30 (6.4)	18 (4.1)	NS
**Symptomatic thrombosis**^f^	16 (1.8)	10 (2.1)	6 (1.4)	NS
**Antibiotic at insertion**^g^	518 (57)	345 (73)	173 (40)	<0.001

CVC, central venous catheter; NS, not significant.

a Numbers are presented as number (%) and continuous variables are presented as median (interquartile range) for the total, before and after the implementation date. Univariate analyses were performed using Mann–Whitney *U* test for continuous variables and χ^2^ test or Fisher’s exact test for categorical variables.

b Bactiguard® Infection Protection (BIP) CVC (Bactiguard AB).

c Non-coated catheter models: Careflow CVC (Merit Medical) or Arrow CVC (Teleflex).

d Changeover wire, anatomic landmark technique and ultrasound guidance only before insertion.

e Catheter malfunction, mispositioning, venous thrombosis and mechanical complications.

f CVC-associated thrombosis causing clinical symptoms requiring diagnostic evaluation or treatment.

g Other than routine prophylaxis against fungal, viral or *Pneumocystis* infections.

**Table II:** Baseline characteristics per patient^a^

Baseline Characteristics per patient	Overall	Before bundle implementation	After bundle implementation	*P*-value
	*N* = 690	*N* = 339	*N* = 351	
**Age, years**	60 [44–69]	58 [40–68]	61 [48–70]	0.010
**Body mass index, kg/m**^2^	25.6 [23–28]	25.7 [23–29]	25.6 [23–29]	NS
Missing	12	4	8	NS
**Biological sex**				
Female	286 (41)	134 (40)	152 (43)	NS
Male	404 (59)	205 (60)	199 (57)	NS
**Main haematological diagnosis**				
AML	256 (37)	151 (45)	105 (30)	<0.001
ALL	75 (11)	43 (13)	32 (9.1)	NS
CML	20 (2.9)	10 (2.9)	10 (2.8)	NS
CLL	25 (3.6)	13 (3.8)	12 (3.4)	NS
Lymphoma	78 (11)	20 (5.9)	58 (17)	<0.001
MDS	34 (4.9)	14 (4.1)	20 (5.7)	NS
MPN	8 (1.2)	2 (0.6)	6 (1.7)	NS
Multiple myeloma	104 (15)	28 (8.3)	76 (22)	<0.001
Other^b^	90 (13)	58 (17)	32 (9.1)	0.003
**Grouped diagnoses**				
Acute myeloid malignancy^c^	290 (42)	165 (49)	125 (36)	<0.001
Other	400 (58)	174 (51)	226 (64)	<0.001
**Splenomegaly**	119 (17)	68 (20)	51 (15)	NS
Missing	29	25	4	<0.001

NS, not significant; AML, acute myeloid leukaemia; ALL, acute lymphoblastic leukaemia; CML, chronic myeloid leukaemia; CLL, chronic lymphocytic leukaemia; MDS, myelodysplastic syndrome; MPN, myeloproliferative neoplasm.

Lymphomas include Hodgkin, non-Hodgkin lymphoma and Waldenström’s macroglobulinaemia.

a Numbers are presented as number (%) and continuous variables are presented as median (interquartile range) for the total, before and after the implementation date. Univariate analyses were performed using Mann-Whitney *U* test for continuous variables and χ-square test or Fisher's exact test for categorical variables.

b Other diagnoses include seminoma and non-seminoma, sickle cell anaemia, Erdheim–Chester disease, aplastic anaemia, multiple sclerosis, hemophagocytic lymphohistiocytosis, myelofibrosis, mixed-phenotype acute leukaemia, acquired haemophilia and plasmacytoma.

c Dichotomized variable comprising AML and MDS.

Unadjusted comparisons of outcomes before vs after the implementation date are presented in Table III. In summary, no differences were observed in the proportions of sCRI, CRBSI or catheter tip colonization. A total of 63 sCRIs were observed during the study period, 30 of 471 CVCs (6.4%) before the implementation date and 33 of 436 (7.6%) after. Similarly, no difference was seen in CRBSI (two cases before vs three cases after the implementation date) or tip colonization, with 22 of 471 cases (4.7%) before and 19 of 436 (4.4%) after. A comparison of the incidence per 1000 catheter-days likewise showed no difference between groups (1.67 vs 2.34; *P* = 0.20 for sCRI and 0.11 vs 0.21; *P* = 0.66 for CRBSI).

**Table III:** Unadjusted comparisons of outcomes before vs after the implementation date

Outcomes	Overall	Before bundle implementation	After bundle implementation	OR	95% CI	*P*-value
	*N* = 907	*N* = 471	*N* = 436			
sCRI	63 (6.9)	30 (6.4)	33 (7.6)	1.20	0.72, 2.02	0.48
CRBSI	5 (0.6)	2 (0.4)	3 (0.7)	1.62	0.27, 12.4	0.60
Catheter tip colonization	41 (4.5)	22 (4.7)	19 (4.4)	0.93	0.49, 1.74	0.82
Missing^a^	467 (51)	141 (30)	326 (75)	-	-	-

Incidence per 1000 catheter-days (Poisson test)	Overall	Before	After	RR	95% CI	*P*-value

sCRI/1000 catheter-days	1.96	1.67	2.34	1.40	0.83, 2.38	0.20
CRBSI/1000 catheter-days	0.16	0.11	0.21	1.91	0.22, 22.8	0.66

sCRI, suspected catheter-related infection; CRBSI, catheter-related bloodstream infection.

a Catheters not sent for analysis.

A multi-variable regression analysis with sCRI as the dependent variable is presented in Table IV. The Hosmer–Lemeshow goodness-of-fit test indicated an adequate model fit (*P* > 0.05). Seven independent variables were selected in the analysis. No variables were independently associated with sCRI, while antibiotics at insertion was associated with a reduced risk (*P* = 0.031). The variable representing before vs after the implementation date was not associated with sCRI.

**Table IV:** Multi-variable logistic regression analysis on sCRI

Independent variables	OR	95% CI	*P*-value
Catheters after implementation date	1.01	0.57, 1.79	0.98
Age, years	0.99	0.97, 1.00	0.15
Male sex	1.35	0.78, 2.41	0.29
Acute myeloid malignancy vs other^a^	1.68	0.96, 2.97	0.07
Catheter dwell time ≥27 days	0.62	0.35, 1.08	0.09
Subclavian vein vs other location^b^	1.26	0.65, 2.64	0.52
Antibiotics at insertion^c^	0.49	0.27, 0.88	0.018

sCRI, suspected catheter-related infection; OR, odds ratio; 95% CI, 95% confidence interval.

a Acute myeloid malignancy is a dichotomized variable comprising acute myeloid leukaemia (AML) and myelodysplastic syndromes (MDS) and is compared with all other diagnoses.

b Dichotomized as the subclavian vein compared with all other veins of insertion.

c Other than routine prophylaxis against fungal, viral or *Pneumocystis* infection.

The micro-organisms isolated from catheter tip cultures and infection episodes are presented in Appendix I.

## Discussion

This observational before-and-after study on 907 CVC insertions in 690 patients with haematological diseases evaluated the impact of a new CVC Anti-infection Double Lumen Bundle on CVC-related infections at our institution. The bundle was not associated with a reduction in sCRI, CRBSI or catheter tip colonization. In multi-variable analysis, no variables were associated with an increased risk of sCRI, whereas antibiotic administration at CVC insertion was associated with a reduced risk of sCRI.

The implementation of the CVC Anti-infection Double Lumen Bundle incorporated a change in dressing type and a new CVC model. Previous studies suggest that chlorhexidine dressings may reduce CVC-related infections in heterogeneous patient populations [8]. However, evidence in patients with haematological diseases is limited and results have been inconsistent. Both a randomized trial in neutropenic patients and a registry-based matched-pair analysis in patients with haematological diseases failed to demonstrate a reduction in CRBSI with chlorhexidine dressings [10,11]. The unchanged occurrence of infections in the present study may therefore reflect limited effectiveness in this population or the influence of other factors, such as increased catheter lumens, change in catheter type or unmeasured time-dependent factors.

The evidence for in-vivo antimicrobial properties of the noble metal alloy–coated BIP CVC is limited, although available studies provide some indication of benefit. A prospective study comparing non-coated catheters and chlorhexidine/silver sulfadiazine–coated CVCs in stem cell transplant recipients demonstrated fewer positive blood cultures, with the coated catheter suggesting a potential protective effect against CVC-related infection [19]. Furthermore, Goldschmidt *et al.* compared non-coated catheters and silver-coated polyurethane CVCs in oncological patients receiving chemotherapy and demonstrated a reduction in catheter-related infections with the coated model (21% vs 10%) [7]. Of note, this study was published before Pronovost *et al.* described simple interventions that markedly reduced CVC-related infection rates and have since been adopted in the majority of centres [20]. The 21% infection rate in the Goldschmidt study is unacceptably high by current standards, and the relevance of the study must be considered as limited [7]. A more recent randomized prospective trial that compared the BIP CVC with uncoated CVCs in immunocompromised patients failed to demonstrate a benefit in reducing CRBSI (8.6% for uncoated catheters vs 13.6% for the BIP CVC catheters) [21]. However, the study included only 45 patients, conferring a high risk of type II error, and its CRBSI definition did not incorporate systemic inflammatory response criteria or exclude alternative causes of infection, both of which are applied in the present study. These discrepancies may partly explain the substantially higher CRBSI incidence reported in that trial compared with our findings. Taken together, the available literature suggests that the BIP CVC at least is no worse than non-coated catheters when it comes to infectious complications.

The final component of the CVC Anti-infection Double Lumen Bundle was the transition from a single-lumen to a double-lumen CVC. An additional lumen allows for parallel infusions and facilitates blood sampling without interrupting ongoing treatments but may theoretically increase infection risk. Assessing the independent effect of catheter lumens is challenging as observational studies are prone to confounding by indication, with more severely ill patients more likely to receive multi-lumen catheters. A meta-analysis reported a slightly higher risk of CRBSI with multiple lumens (OR: 2.15; 95% CI: 1.00–4.66), although this association weakened after adjustment for baseline differences [22]. Another systematic review reported similar findings (OR: 2.58; 95% CI: 1.24–5.37) [23], while neither review found differences in catheter tip colonization. Overall, the evidence does not firmly support that multiple lumens increase CVC-related infection risk. Catheter lumens were not included as an independent variable due to collinearity with the implementation date.

Subclavian insertion has previously been associated with a lower risk of CVC-related infections than the jugular vein [24]. In our cohort, subclavian catheterization was not associated with sCRI, although insertion practices changed after bundle implementation: subclavian insertions decreased (86% vs 72%; *P* < 0.001), while internal jugular insertions increased (14% vs 27%; *P* < 0.001). The reduced use of the subclavian vein may reflect preference for the compressible internal jugular vein, particularly in patients with higher perceived bleeding risk. The shift in preferred insertion side likely reflects increased adoption of supraclavicular guidewire visualization during right-sided CVC insertion [25,26].

The median catheter dwell time in the present study (28 and 25 days) may appear long for non-tunnelled CVCs. In most settings, non-tunnelled catheters are intended for short-term use, and infection risk has been reported to increase with prolonged catheter dwell time [27]. However, interpretation of associations between dwell time and infection risk in observational studies is complicated by potential reverse causality as catheters that develop infection are often removed earlier. Current guidelines recommend that CVCs should be removed when no longer clinically indicated rather than replaced solely based on time [28,29].

Antibiotic exposure at CVC insertion was associated with sCRI; however, this finding should be interpreted with caution given the study design and the definition of exposure. Routine single-dose rifampicin prophylaxis prior to CVC insertion was discontinued in 2019, and residual confounding with the pre-implementation cohort cannot be excluded. Furthermore, antibiotic exposure was defined as any ongoing systemic antibiotic treatment at catheter placement and was not limited to procedural prophylaxis, which may also have influenced the observed association. Taken together, the association between antibiotic exposure and sCRI is more likely explained by residual confounding than by a causal effect of antibiotics.

### Strengths and limitations

This study has several strengths. First, it includes a large, real-world cohort of 907 CVC insertions in 690 patients with haematological diseases, providing a robust and comprehensive dataset rarely available in this population. Second, the use of detailed EHR extraction combined with manual verification ensures high data accuracy, while adherence to standardized and predefined infection definitions strengthens internal validity. Third, the study evaluates a multi-faceted, practice-changing intervention under real-life clinical conditions, offering highly relevant evidence for institutions considering similar infection prevention strategies.

The before-and-after design is a major limitation as time-dependent potential confounding factors cannot be ruled out. In line with this, and to avoid masking of any differences in before vs after cohorts in baseline variables, there was no correction for multiple testing. This approach ensures that any meaningful trends or differences in baseline variables were not overlooked. However, it should be observed that the potential downside of this is an increased likelihood of type I errors.

The risk of missing data due to the retrospective design is another limitation. Despite strong adherence to documentation of CVC insertion at the hospital, there was no way of finding non-documented catheters. Furthermore, it cannot be guaranteed that all complications were registered. In addition, catheter tip cultures were obtained only when infection was clinically suspected, which may have limited the detection of asymptomatic catheter colonization. Severe complications may have been reported more frequently than minor complications due to clinicians’ documentation practices. There is also a risk of missing or misinterpreting complications during EHR review, although this was minimized through joint assessment. While a randomized controlled trial would have been the ideal design, the simultaneous implementation of several routine changes made a retrospective cohort study most suitable. Causality cannot be inferred from this observational design.

Due to the limited number of events, logistic regression analysis could not be performed for CRBSI; therefore, sCRI was used to control for confounders. To avoid model overfitting, only a limited number of variables were included in the multi-variable analysis, leaving other potential predictors unexplored.

Another limitation is the lack of uniform definitions of catheter-related infections, including catheter tip colonization, sCRI and CRBSI, which limits comparability between studies and complicates interpretation of the evidence.

In conclusion, no differences in sCRI, CRBSI or catheter colonization were observed when comparing CVC insertions before and after introduction of the CVC Anti-infection Double Lumen Bundle. None of the selected variables were associated with an increased risk of sCRI, whereas antibiotic administration at CVC insertion was associated with a reduced risk.

These findings may contribute to the refinement of clinical routines and protocols supporting evidence-based care and patient safety. Further studies are needed to determine the most appropriate CVC model and dressing type for patients with haematological diseases.

## CRediT authorship contribution statement

**M.L. Lindblad:** Writing – original draft, Project administration, Investigation, Formal analysis, Data curation. **E. Pålsson:** Writing – review & editing, Investigation, Data curation. **V. Lazarevic:** Writing – review & editing, Supervision, Methodology, Investigation. **O. Borgquist:** Writing – review & editing, Supervision, Methodology, Conceptualization. **T. Kander:** Writing – review & editing, Supervision, Project administration, Methodology, Investigation, Funding acquisition, Conceptualization.

## Funding sources

This study was funded by the Swedish government grant for clinical research within the Swedish National Health Service (ALF Grant number: 748404) (T.K.), Regional Research Grant from Region Skåne, Sweden (T.K.), LÖF, the Swedish mutual insurance company owned by the regions (T.K.) and The Swedish Society of Medicine, Sweden (T.K.).

## Conflict of interest statement

None declared.
