# Supplementary material for: Impact of an Anti-infection Double Lumen Bundle on the incidence of central venous catheter–related infections in haematological patients: a retrospective cohort study
Source: Infect Prev Pract. 2026 Mar 30;8(2):100536. doi: 10.1016/j.infpip.2026.100536 (PMC13127321; doi:10.1016/j.infpip.2026.100536)
Supplement: Supplementary file 1 — Multimedia component 1 [file mmc1.docx]

Appendix I

**Isolated microorganisms per outcome^[[1]](#endnote-1)^**

| **Organism** | **sCRI  N = 66** | **CRBSI  N = 9** | **Colonized tips  N = 43** |
| --- | --- | --- | --- |
| **Gram positives** |  |  |  |
| Staphylococcus |  |  |  |
| Coagulase negative |  |  |  |
| S. epidermidis | 43 (65) | 7 (78) | 34 (79) |
| S. capitis | 1 (1.5) | 0 (0.0) | 0 (0.0) |
| S. haemolyticus | 2 (3.0) | 0 (0.0) | 1 (2.3) |
| S. lugdunensis | 1 (1.5) | 0 (0.0) | 0 (0.0) |
| Other | 4 (6.1) | 0 (0.0) | 2 (4.7) |
| Staphylococcus aureus | 4 (6.1) | 2 (22) | 3 (7.0) |
| Streptococcus mitis | 2 (3.0) | 0 (0.0) | 0 (0.0) |
| Enterococcus faecalis | 1 (1.5) | 0 (0.0) | 1 (2.3) |
| **Gram negatives** |  |  |  |
| Escherichia coli | 1 (1.5) | 0 (0.0) | 0 (0.0) |
| Klebsiella pneumoniae | 1 (1.5) | 0 (0.0) | 0 (0.0) |
| Pseudomonas aeruginosa | 1 (1.5) | 0 (0.0) | 0 (0.0) |
| **Yeasts** |  |  |  |
| Candida crusei | 2 (3.0) | 0 (0.0) | 1 (2.3) |
| **Unknown organism^[[2]](#endnote-2)^** | 3 (4.5) | 0 (0.0) | 1 (2.3) |

1. Registering of multiple organisms was possible. Abbreviations: sCRI, Suspected Catheter-related Infection; CRBSI, Catheter-related Bloodstream Infection. [↑](#endnote-ref-1)
2. Culture data has been archived and is unavailable. [↑](#endnote-ref-2)
